# Does Social Stigma and Neglect Post-COVID-19 Matter? A Case Report on Brief Psychotic Disorder Post-COVID-19 and Self-Quarantine

**DOI:** 10.7759/cureus.12973

**Published:** 2021-01-28

**Authors:** Nischit Baral, Govinda Adhikari, Sandip Karki, Andrew Champine, Parul Sud

**Affiliations:** 1 Internal Medicine, McLaren Flint/Michigan State University, Flint, USA; 2 Psychiatry, McLaren Flint/Michigan State University, Flint, USA

**Keywords:** covid-19, self-quarantine, brief psychotic disorder, social stigma, neglect

## Abstract

Social stigma and neglect post-coronavirus disease 2019 (COVID-19) and self-quarantine can be associated with a brief psychotic disorder (BPD). A 53-year-old African-American man with no significant past medical and psychiatric history was brought to the emergency department (ED) with symptoms of persecutory delusions post COVID-19 and self-quarantine. His symptoms included false beliefs that people were plotting to kill him which made him combative at work and home. As his symptoms worsened, his wife brought him to the hospital. He was given intramuscular haloperidol 5 mg one dose in the ED. The Clinical Health Psychology and Psychiatry team diagnosed the patient with BPD as per the Diagnostic and Statistical Method of Mental Disorder Fifth Edition (DSM-5). Over the next few days, his symptoms slowly improved. At follow-up visit in the outpatient clinic in a week, we found him back to his baseline without any delusional thoughts. Increased stressors post COVID-19, neglect at home, and social stigmata at work associated with COVID-19 along with his individual vulnerability appeared to be the cause of his delusions but various other mechanisms may exist. Our case raises the question: does social stigma and neglect post-COVID-19 and self-quarantine matter?

## Introduction

The novel coronavirus disease 2019 (COVID-19) is responsible for a pandemic that has become a global health problem in the world. As per the most recent report of the Centre for Disease Control (CDC) during the development of this manuscript, since January 21, 2020, more than 22 million cases of COVID have been diagnosed and the death rate estimated to be 111 per 100,000 people in the US alone [1]. The symptoms of COVID-19 are widely variable ranging from pulmonary to extra-pulmonary manifestations like cardiovascular, gastrointestinal, neurological, and thromboembolic [2]. We report a case of a 53-year-old African-American male with no previous psychiatric history who presented with symptoms of delusions and combativeness post-COVID-19 and self-quarantine. The unique qualities we wish to explicate demonstrate the social stigma elements and spontaneous remission of psychiatric symptoms related to COVID-19.

## Case presentation

On October 24, 2020, a 53-year-old African-American male with no significant past medical history and no neuropsychiatric history was tested positive for COVID-19 with severe acute respiratory syndrome coronavirus 2 (SARS-CoV-2) polymerase chain reaction (PCR) test in an urgent care center. He had visited an urgent care for symptoms of fever, cough and, weakness for two days. These symptoms improved on their own during 14 days of self-quarantine. There were no acute events like psychosis, substance abuse, depression, or suicidal ideation during the self-isolation. The patient was not treated with any medication for COVID-19. After five weeks of COVID-19 diagnosis, he experienced delusional thoughts of people plotting to kill him. He thought his wife was trying to poison him at home. He became combative. His delusions and combativeness worsened with time so his wife brought him to the emergency department (ED) of a nearby hospital (Hospital A) on December 13, 2020. In Hospital A, the patient started to believe that his wife wanted him to get COVID-19. Her motive was to have him die in hospital. During his care, the patient became increasingly suspicious in Hospital A and he left against medical advice. The patient was brought to our academic community hospital (Hospital B) the next day at 1:34 am by his wife. He continued to be delusional and combative. He was given intramuscular haloperidol 5 mg one dose in the emergency room (ER). His wife explained that he was a different man before. At baseline, the patient was reported to be respectful, friendly, and a supportive husband. His daughter corroborated these statements. We consulted the Behavioral Health team, which is composed of the consultation liaison (CL) service staffed by clinical health psychology and psychiatry. The evaluation of the CL team provided the diagnosis of brief psychotic disorder (BPD). The CL team counseled the patient regarding his symptoms and taught him coping strategies. During the next few days, he had improvement in his symptoms. However, the patient left against medical advice from our Hospital B. He had an outpatient clinic visit with us in seven days. During the visit, we found him back to his baseline without any delusional symptoms. 

Mental status evaluation

The patient was alert and oriented to time, situation and place. His mood was “annoyed”, affect was irritable. He denied any suicidal ideation or homicidal ideation in the past or present which was confirmed by the patient’s wife. He denied any hallucinations in the past or present. There appeared to be some evidence of delusions given conflicting reports between what the patient was stating and the history that the wife was reporting. His thought process was linear but somewhat perseverative on his conflict with his wife and financial troubles. Speech was very low in volume, with some potential word intrusions. The patient evidenced poor insight and judgment. The patient was not sure why he was admitted to the hospital. He reported recent poor sleep which he attributed to stress at work. He felt stigmatized of getting COVID-19 at work. He felt neglect at home. He reported his appetite as unchanged. He did not express any desire for treatment personally. The wife offered him melatonin gum, but the patient stated that she was trying to poison him. He denied any mismanagement of finances by him or his wife. The remaining physical examinations were unremarkable other than the mental status exam as detailed above.

Investigations

Initially in the ER, an electrocardiogram (EKG), chest X-ray, complete metabolic panel (CMP), complete blood count (CBC), and lactic acid were within normal limits. Blood alcohol level was zero mg/dl. Venous ammonia level was within normal limits. Urine drug screen (UDS) was negative. The patient underwent a CT scan of the head which was normal. SARS-CoV-2 rapid PCR test on December 14, 2020 was negative. Rapid plasma reagin test was negative. Human immunodeficiency virus type-1 (HIV-1) antibody, human immunodeficiency virus type-2 (HIV-2) antibody, and p24 antigen were non-reactive. Serum inflammatory markers were ordered including serum lactate dehydrogenase, ferritin, and D-dimer. Only serum ferritin was mildly elevated with a level of 400.9 ng/ml. His thyroid-stimulating hormone, urinalysis, troponin-1 high sensitivity, prothrombin time, activated partial thromboplastin time, international normalized ratio, liver panel, vitamin B12, and folate were all within normal limits. 

Differential diagnosis

The differentials included schizophrenia spectrum and other psychotic disorder, personality disorder, BPD due to substance use or medical condition, and delirium. DSM-5 diagnostic criteria for BPD requires a sudden onset of a psychotic episode, one or more psychotic symptoms which include delusions, hallucinations or disorganized speech, duration of more than one day but less than one-month, eventual full return to premorbid level of functioning, and the disorder is not better explained by alternative diagnosis. We ruled out other differential diagnoses based on the timeline of his symptoms for less than a month; there were no symptoms other than delusions. Normal workup for UDS, normal labs and imaging, and no previous history of psychiatric illness or substance abuse was noted.

Treatment

On arrival at the ED, the patient was aggressive and combative so we had to give him a dose of intramuscular haloperidol 5 mg once. His vitals were stable; CBC, CMP, UDS, and EKG were within normal limits. He was transferred to the medical floor after assessment from the CL team.

Outcome and follow up

Following the psychiatric evaluation, counseling, and treatment with one dose of intramuscular haloperidol 5 mg, his delusional symptoms improved within 24 hours. He was more responsive and interactive. He gained insight into the condition. He was fearful of the social stigma of being COVID-19 positive. The patient left the hospital against medical advice. After five days, we talked with the patient and his wife who agreed to follow up with us in the clinic within the next two days. In the clinic, on mental state examination, we found that he had gained insight into his delusions post-COVID-19. He had no delusional symptoms. He was back to his work. He reported lack of stigmatization in his job and felt no neglect at home.

## Discussion

The functional receptor for coronaviruses is angiotensin-converting enzyme-2 (ACE-2) and the infection is triggered by binding of the spike protein of the virus to ACE2 [2]. However, SARS-CoV-2 is a neurotropic virus and can invade the central nervous system [2,3]. COVID-19 causes a hypercoagulable state and can cause microvascular infarcts due to thromboembolism in the brain [4,5]. It can cause neuroinflammation, which subsequently can cause neuropsychiatric manifestations like acute psychosis, delusions and, other neurocognitive symptoms [3-5]. Furthermore, treatment with high dose corticosteroids for days can be associated with neuropsychiatric manifestations of the disease [2-6].

The exact mechanism by which COVID-19 causes neuropsychiatric symptoms is not clear to date. Cytokine dysregulation, post-infectious autoimmunity, direct viral neuroinvasion and neuroinflammation and, transmigration of peripheral myeloid cells has been described as potential mechanisms of neuropsychiatric symptoms in COVID-19 [7]. Furthermore, self-isolation, infection, and associated psychosocial stressors such as financial burden and job insecurity secondary to the pandemic can contribute significantly to psychotic symptoms, especially in vulnerable people. Steroids frequently used in the treatment of severe COVID-19 infection may be another potential cause of psychosis in such patients [2,8,9]. 

Stable patients infected with COVID-19 infection are recommended a period of self-quarantine. The self-isolation during this period can play a major role in the development of neuropsychiatric illness especially in vulnerable individuals [10].

In our patient, we do not know the exact cause of psychosis but in view of symptom onset after five weeks of being diagnosed with COVID-19, the role of stress of contracting COVID-19 and its social stigma, self-isolation, and neglect at home and work or post-infectious autoimmunity after COVID-19 are possible in our case. 

We have included the three studies from the literature search which describe psychotic symptoms associated with COVID-19. In the study by Smith et al., the patient developed symptoms of BPD after four days of onset of symptoms of COVID-19 [11]. In another report by Haddad et al., the patient developed BPD after seven days of being diagnosed with COVID-19 [8]. The case series in Italy also showed BPD in patients during the COVID-19 pandemic [12]. The study by Anmella et al. is also unique in that it developed in a health care worker who was caring for COVID-19 patients [13]. These studies are summarized in Table 1.

**Table 1:** Describing a brief synopsis of important features of reported cases COVID-19: coronavirus disease 2019

Study	Demographics	Disease onset	Symptoms	Treatment	Outcome
Smith et al. [11]	36-year Afro-American lady, no known psychiatric history	4 days after onset of upper respiratory symptoms (Rhinorrhea and nasal congestion)	Rapidly progressive change in behavior, persecutory delusion, anxiety	Initially treated with olanzapine for 7 days and clonazepam for anxiety.	Complete recovery was noted on Follow up after 1 week from discharge.
Haddad et al. [8]	30-year male from South-east Asia working in Qatar	Body aches and anxiety for 4 days prior to testing positive for COVID-19*.	Paranoid delusions, increased agitation, anxiety, insomnia, and restlessness	Risperidone 1 mg bid and mirtazapine 30mg/day.	Follow up after 2 weeks showed complete resolution of symptoms with the patient being able to return to work.
Anmella et al. [13]	42-year female, physician, no known medical, psychiatric or family history.	2 weeks before caring for patients with suspicion of COVID-19 and psychiatric symptoms.	Delusional ideas of catastrophe regarding the current pandemic situation suspiciousness and delusions of self-reference, surveillance and persecution, with high affective and behavioral involvement	Olanzapine 10 mg	Patient symptoms improved with the olanzapine.

## Conclusions

Our patient reported that he had never experienced delusional symptoms before. He believed that the acute stressors post COVID-19 including social stigma of being COVID-19 positive at work and neglect at home during self-isolation might have caused him to get delusional thoughts. The unique aspect of our case report is that our patient had returned to work post-quarantine and developed delusions five weeks post COVID-19 diagnosis. Our case highlights that BPD can occur post-COVID-19 with increased stressors being one of the major etiologic factors. This psychotic period in our patient was brief and resolved quickly. Social stigma and neglect post-COVID-19 matters. Social and emotional support play a major role in the recovery of such patients rather than pharmacotherapy alone.
